# Outcomes of laparoscopic transabdominal preperitoneal hernia repair in the elderly population: a retrospective cohort study

**DOI:** 10.20452/wiitm.2024.17899

**Published:** 2024-09-20

**Authors:** Dawid Golik, Krzysztof Jędras, Przemysław Sroczyński, Grzegorz Dobkowski, Michał R. Janik

**Affiliations:** General Surgery Department, Military Institute of Aviation Medicine, Warszawa, Poland; General Surgery Department, Hospital of Our Lady of Perpetual Help, Wołomin, Poland

**Keywords:** elderly, hernia repair, laparoscopic surgery, outcomes, transabdominal preperitoneal hernia repair

## Abstract

**INTRODUCTION:**

Hernia repair is a common procedure, especially among the elderly. In the face of rising life expectancy, adequate hernia management in older adults is crucial. Laparoscopic transabdominal preperitoneal patch plasty (TAPP) for hernia repair is minimally invasive, but poses challenges in the elderly due to a larger number of comorbidities.

**AIM:**

The aim of this study was to evaluate the safety and efficacy of TAPP hernia repair in patients aged 70 years and older.

**MATERIALS AND METHODS:**

Our retrospective study reviewed data of patients who underwent elective unilateral TAPP repair for primary inguinal hernia between September 2021 and December 2023. The patients were divided by age: 70 and older (cases) and younger than 70 (controls). Primary outcomes included recurrence rate, surgical site infections (SSIs), operative time, and hospital stay. Data were analyzed with descriptive statistics and inferential tests.

**RESULTS:**

The study included 201 patients (47 cases and 154 controls). The mean (SD) age was 75.74 (4.73) years for the cases and 53.47 (12.8) years for the controls. No recurrences were found in the elderly (cases) group, while the control group recorded a 3.92% recurrence rate (*P *= 0.34). SSIs affected 2.13% of the cases and 1.31% of the controls (*P *= 0.55). Operative times were similar (71.44 vs 71.96 min; *P *= 0.8)*.* Hospital stay was 3.11 days for the cases and 3.04 days for the controls (*P *= 0.14).

**CONCLUSIONS:**

Laparoscopic TAPP hernia repair is a safe and effective procedure in the elderly and its outcomes are comparable to those recorded in younger patients. Further studies are needed to validate these results.

## INTRODUCTION

Hernia repair is one of the most common surgical procedures performed worldwide, and a significant proportion of patients subjected to it are elderly. As life expectancy continues to rise, the prevalence of hernias in older adults is increasing, making hernia management in this demographic group an important area of clinical focus.^1^

The study demonstrates that unilateral open mesh repair significantly facilitates pain control and improves quality of life in patients with inguinal hernias. Most patients reported satisfactory physical and emotional postsurgery condition, highlighting the procedure’s effectiveness and the value of using SF-36 questionnaire for assessing patient outcomes.^2^ However, laparoscopic transabdominal preperitoneal patch plasty (TAPP) for hernia repair has gained popularity due to its minimally invasive nature, reduced postoperative pain, and shorter recovery time, as compared with open surgical techniques.^3^^; ^^4^^; ^^5^^; ^^6^ Despite these advantages, the elderly population presents unique challenges, including higher rates of comorbidities and increased susceptibility to surgical complications.^1^ Previous studies have demonstrated the efficacy of TAPP hernia repair in the general population, but there are limited data specifically addressing outcomes in elderly patients, particularly those over the age of 70.^1^^; ^^7^^; ^^8^

Understanding the outcomes of TAPP hernia surgery in patients aged 70 years and older is crucial for optimizing perioperative care and improving surgical outcomes. This demographic group often shows different physiological responses to surgery and faces a higher risk of postoperative complications, which necessitates tailored surgical approaches and postoperative management strategies.

## AIM

The aim of this study was to evaluate the safety and efficacy of laparoscopic TAPP hernia repair in the population aged 70 years and older. We hypothesized that laparoscopic TAPP hernia repair could be performed safely in this group of patients, with outcomes comparable to those observed in younger populations, provided that appropriate perioperative care was ensured.

## MATERIALS AND METHODS

The study was conducted as a retrospective observational analysis at our surgical center. We reviewed the clinical data of patients who underwent laparoscopic TAPP inguinal hernia repair between September 2021 and December 2023. The study adhered to the STROBE guidelines for observational studies.^9^

We included patients who were 18 years or older and underwent elective unilateral TAPP repair for primary inguinal hernia. The study excluded patients who underwent a bilateral inguinal hernia repair, open inguinal hernia repair, emergency hernia repair, or cases where no sufficient follow-up data were available. The participants were selected from the hospital’s electronic medical records. Follow-up was conducted through routine postoperative visits and review of medical records up to 1 year postsurgery. The patients were divided into 2 groups based on their age: the case group which included patients aged 70 years and older and the control group which comprised participants below 70 years.

No additional matching criteria were applied, as this was not a matched study.

TAPP inguinal hernia repair was performed under general anesthesia. Pneumoperitoneum was established and 3 trocars were inserted in a standard configuration. The peritoneum was incised to access the preperitoneal space, where the hernia sac was identified and reduced. A synthetic mesh with a minimum size of 15 cm × 10 cm was placed to cover the hernia defect, ensuring it extended at least 3 cm beyond the defect margins, which is the minimum overlap recommended in the literature.^10^^; ^^11^^; ^^12^^; ^^13^ For hernias classified as M3 or L3 according to the European Hernia Society (EHS) classification, the mesh was secured with glue or sutures.^14^^; ^^15^^; ^^16^ Our technique for mesh fixation in M3 and L3 inguinal hernias goes beyond the EHS recommendations that advise mesh fixation only for M3 hernias.^17^ The peritoneum was then closed over the mesh to restore the anatomy. Operative time, postoperative complications, and patient recovery were meticulously recorded.

The primary outcomes assessed in this study included: 1) recurrence rate, that is, the incidence of hernia recurrence identified through clinical examination or imaging, within 1 year postsurgery; 2) surgical site infections (SSIs) diagnosed based on clinical signs or microbiological evidence, occurring within 180 days postsurgery; 3) operative time, that is, the duration of the surgery, recorded in minutes from the initial incision to the completion of the procedure; and 4) length of hospital stay, that is, the total number of days from the day of surgery to the day of discharge from the hospital.

Data for this study were obtained from electronic medical records. Body mass index (BMI) expressed in kg/m^2^ was calculated using standard height and weight measurements recorded during preoperative assessments. Operative time, length of hospital stay, and recurrence rates were directly extracted from surgical and follow-up records. SSIs were diagnosed based on established clinical criteria and documented in postoperative notes.

This study may be subject to several potential biases, including selection bias due to the retrospective nature of data collection, recall bias resulting from reliance on medical records, and observer bias in the diagnosis of SSIs. Additionally, the lack of randomization and matching criteria might introduce confounding variables that could affect the outcomes.

The sample size for this study was determined by the number of eligible patients who underwent laparoscopic TAPP inguinal hernia repair at our surgical center between September 2021 and December 2023. The total number of participants included in the analysis was 201, providing a sufficient cohort to assess the primary outcomes of recurrence rates, SSIs, operative time, and length of hospital stay.

Data analysis was conducted using SAS Studio statistical software (SAS Institute Inc., Cary, North Carolina, United States). Quantitative variables (operative time, length of hospital stay) were handled using means and SD for continuous variables. Groupings were based on age to enable comparison between the elderly and nonelderly patients.

Descriptive statistics, including means, SD, and percentages were calculated for demographic and operative variables. Categorical variables were compared between the age groups using the χ^2^ test or the Fisher exact test. The Wilcoxon signed-rank test was employed to compare continuous variables. Statistical significance was determined at a *P* value below 0.05.

This retrospective study used fully anonymized medical data, ensuring that no patients could be identified. Consequently, approval from the institutional ethics committee was not required. Patient confidentiality and data privacy were strictly maintained, and informed consent was waived due to the retrospective nature of the analysis.

## RESULTS 

We reviewed and analyzed 201 patients who underwent TAPP inguinal hernia repair. Their basic characteristics are presented in TABLE 1. Among these, 47 patients (23.38%) were classified as elderly (cases), while the remaining 154 (76.62%) served as controls. The mean (SD) age in the case group was 75.74 (4.73) years, while in the control group it was 53.47 (12.8) years. The mean (SD) weight of the patients in the case group was 77.34 (10.63) kg, as compared with 83.19 (14.27) kg in the control group. The mean (SD) height was 174.04 (8.12) cm for the case group and 176.02 (9.44) for the controls. The mean (SD) BMI was 25.47 (2.65) kg/m² in the case group and 26.83 (4.18) kg/m² in the control group. Right-sided hernias were identified in 53.19% of the elderly patients and 57.79% of the controls. Approximately 89.36% of case group patients were men, as compared with 88.96% in the control group. TABLE 2 presents the distribution of hernia types according to the EHS classification.

**TABLE 1:** Baseline characteristics of the study participants

Variable	Cases (n = 47)		Controls (n = 154)	
	Mean	SD	Mean	SD
Age, y	75.74	4.73	53.47	12.8
Weight, kg^a^	77.34	10.63	83.19	14.27
Height, cm	174.04	8.12	176.02	9.44
BMI, kg/m^2a^	25.47	2.65	26.83	4.18
Right side, %	53.19	–	57.79	–
Men, %	89.36	–	88.96	–

a *P* <0.05

Abbreviations: BMI, body mass index

**TABLE 2:** Inguinal hernia location in the study participants according to the European Hernia Society classification

Variable	All participants, %	Cases, %	Controls, %
Medial	36.6	11.1	43.7
1	6.67	–	7.14
2	26.67	100	21.43
3	66.67	–	71.43
Lateral	58.5	88.9	50
1	20.83	–	31.25
2	62.5	62.5	62.5
3	16.67	37.5	6.25
Femoral	4.8	–	3.1
1	100	–	100
2	–	–	–
3	–	–	–

There were no recurrences within 1 year postsurgery in the elderly group, whereas in the control group, the recurrence rate was 3.92% (*P* = 0.34). The incidence of SSIs was 2.13% in the elderly group and 1.31% in the control group, with the difference not being significant. All observed SSIs were superficial wound infections. The mean (SD) operative time for the elderly group was 71.44 (28.77) minutes, and 71.96 (25.93) minutes for the control group (*P *= 0.8). The mean (SD) length of hospital stay was 3.11 (0.29) days for the elderly group, and 3.04 (0.31) days for the controls (*P *= 0.14). The findings for the elderly and control groups are summarized in TABLE 2. The post hoc logistic regression analysis revealed no significant effects of age (*P *= 0.35) or BMI (*P *= 0.65) on the outcomes, with odds ratios of 0.98 (95% CI, 0.93–1.03) and 0.95 (95% CI, 0.76–1.19), respectively. Our model demonstrated a moderate discriminative ability with a C-statistic of 0.649.

## DISCUSSION

The study indicated no significant differences in postoperative outcomes between the elderly patients (aged 70 years and older) and the control group patients (younger than 70 years) who underwent laparoscopic TAPP inguinal hernia repair. Specifically, the recurrence rate within 1 year postsurgery was 0% for the elderly group, and 3.92% for the control group, a difference that did not reach statistical significance. Similarly, the SSI rates were comparable between both groups, indicating no significant disparity. Operative times were also similar, with insignificant difference in mean duration. Furthermore, the length of hospital stay showed only a marginal, nonsignificant difference, with the elderly group averaging 3.11 days and the control group 3.04 days. The logistic regression analysis confirmed that neither age nor BMI had a significant impact on the recurrence rate.

These results suggest that laparoscopic TAPP hernia repair is a viable and safe option for elderly patients, with outcomes comparable to those observed in the younger population. The moderate discriminative ability of the logistic regression model, as indicated by the C-statistic of 0.649, supports the reliability of these findings. Future research could benefit from larger sample sizes and prospective study designs to further validate these results and explore additional factors that might influence surgical outcomes in different age groups.

This study contributes to the ongoing debate on the appropriateness and safety of laparoscopic TAPP hernia repair in elderly patients, particularly those aged 70 years and older. The nationwide prevalence study highlights a bimodal age distribution for inguinal hernia repair, peaking in early childhood (0–5 years) and old age (75–80 years).^18^ This demographic trend underscores the necessity of evaluating surgical approaches in elderly patients who represent a significant portion of the hernia repair population.

Elective hernia surgery aims to improve symptoms and prevent acute surgical emergencies such as incarceration or strangulation. They are particularly concerning in the elderly, as emergency repairs are associated with significantly higher morbidity and mortality rates, as compared with elective procedures.^19^ Despite these risks, there is still uncertainty regarding the appropriateness of elective intervention in elderly patients with minimal symptoms and comorbidities, as the benefits must be weighed against the surgical risks.

Our findings align with those of the Swedish and Danish Hernia Registries, which reported a marked increase in postoperative complications for both laparoscopic and open preperitoneal procedures in patients older than 65 years.^20^^; ^^21^ This suggests that age is a critical factor influencing postoperative outcomes, regardless of the surgical technique employed. However, it is important to note that our study found no significant differences in postoperative outcomes, including recurrence rates and SSIs, between the elderly patients and the younger controls. This discrepancy highlights the need for more nuanced research to identify specific patient characteristics that may predict better or worse outcomes in the elderly populations.

Existing literature presents mixed results regarding the safety and efficacy of laparoscopic versus open inguinal hernia repair in the elderly. Retrospective comparative studies involving patients aged 80 years and older have shown no significant differences in perioperative outcomes between open and laparoscopic / endoscopic approaches.^22^^; ^^23^ This suggests that with proper patient selection and surgical expertise, laparoscopic TAPP repair can be a safe and effective option for elderly patients.

Our study supports laparoscopic TAPP hernia repair as a viable option for elderly patients, with outcomes comparable to those observed in younger populations. Nonetheless, the lack of significant differences in our study underlines the importance of individualized patient assessment and consideration of comorbidities when planning elective hernia repairs in elderly patients.

This study has several limitations to consider when interpreting the results. First, the retrospective design carries inherent risks of selection bias and incomplete data, as it relies on the accuracy and completeness of medical records. Second, the study was conducted at a single surgical center, which may limit the generalizability of the findings to other settings with different patient demographics or surgical practices. Third, although we adjusted the groups for age and BMI, other potential confounding factors, such as comorbidities, surgical technique variations, and surgeon experience were not considered, which could have affected the outcomes. Additionally, the relatively small sample size may limit the statistical power to detect differences between groups and generalize the findings. Even though metrical age is often used as a benchmark, it does not necessarily reflect a person’s true health status. Biological age, which considers factors such as organ function and overall physical condition, provides a more accurate picture of an individual’s health. Finally, the follow-up period of 1 year may not be sufficient to capture late recurrences or long-term complications, necessitating further studies with longer follow-ups to fully understand the long-term outcomes of laparoscopic TAPP hernia repair in elderly patients.

## CONCLUSIONS

This study demonstrates that laparoscopic TAPP hernia repair is a viable and safe option for patients aged 70 years and older, yielding outcomes that are comparable to those observed in younger patients. The analysis found no significant differences in recurrence rate, SSIs, surgery duration, or hospital stay between the older and younger groups, suggesting that advanced age should not be a contraindication to laparoscopic TAPP repair. These findings underscore the necessity of individualized patient assessment, particularly in elderly individuals who may suffer from additional conditions or factors that could impact surgical outcomes. This study adds a valuable insight to the ongoing debate regarding the appropriateness of laparoscopic hernia repair in older adults, a demographic group that is growing due to increasing life expectancy.
